# Resting-State fMRI Whole Brain Network Function Plasticity Analysis in Attention Deficit Hyperactivity Disorder

**DOI:** 10.1155/2022/4714763

**Published:** 2022-09-26

**Authors:** Yi Tang, Shuxing Zheng, Yin Tian

**Affiliations:** ^1^Bio-Information College, Chongqing University of Posts and Telecommunications, Chongqing 400065, China; ^2^College of Computer Science and Technology, Chongqing University of Posts and Telecommunications, Chongqing 400065, China

## Abstract

Attention deficit hyperactivity disorder (ADHD) is a common mental disorder in children, which is related to inattention and hyperactivity. These symptoms are associated with abnormal interactions of brain networks. We used resting-state functional magnetic resonance imaging (rs-fMRI) based on the graph theory to explore the topology property changes of brain networks between an ADHD group and a normal group. The more refined AAL_1024 atlas was used to construct the functional networks with high nodal resolution, for detecting more subtle changes in brain regions and differences among groups. We compared altered topology properties of brain network between the groups from multilevel, mainly including modularity at mesolevel. Specifically, we analyzed the similarities and differences of module compositions between the two groups. The results found that the ADHD group showed stronger economic small-world network property, while the clustering coefficient was significantly lower than the normal group; the frontal and occipital lobes showed smaller node degree and global efficiency between disease statuses. The modularity results also showed that the module number of the ADHD group decreased, and the ADHD group had short-range overconnectivity within module and long-range underconnectivity between modules. Moreover, modules containing long-range connections between the frontal and occipital lobes disappeared, indicating that there was lack of top-down control information between the executive control region and the visual processing region in the ADHD group. Our results suggested that these abnormal regions were related to executive control and attention deficit of ADHD patients. These findings helped to better understand how brain function correlates with the ADHD symptoms and complement the fewer modularity elaboration of ADHD research.

## 1. Introduction

Attention deficit hyperactivity disorder (ADHD) is a neurodevelopmental disorder mostly in childhood, which is characterized as inattention, hyperactivity, and inappropriate impulsiveness [1]. Reviews about ADHD surveyed the samples of children and adolescents from 35 countries around the world and estimated the prevalence of ADHD at 5.29% [2–4]. ADHD not only affects learning development, healthy growth, and daily life but also brings tremendous pressure to the family. Accordingly, exploring the causes of ADHD and relevant cognitive neural mechanisms could help in the detection and treatment of the disease.

Recently, neuroimaging techniques have been widely used to elucidate the pathophysiology of ADHD, especially functional magnetic resonance imaging (fMRI) which has made progress to estimate the brain function of ADHD patients due to its high spatial resolution. But the use of resting-state fMRI (rs-fMRI) or task-state fMRI has always been a controversial issue. In fact, the brain consumes a lot of energy at rest, showing spontaneous neural activity, whereas the increase of the brain energy during the task mode is insignificant (<5%) [5]. Moreover, the disturbances of spontaneous neural activity in the brain have been reported being associated with psychiatric disorders including ADHD. These pathological disorders of intrinsic activity show good separation between healthy and patient individuals, suggesting that using rs-fMRI to study spontaneous neural activity may contain valuable diagnostic information [6]. Furthermore, rs-fMRI usually requires participants to open or close their eyes without involving any complex tasks. Considering that it is difficult for ADHD children to successfully complete complex experiment tasks, using rs-fMRI is more beneficial than the task mode.

Early rs-fMRI ADHD researches focused on aberrant changes of independent brain regions or between different brain regions. Zang et al. found that ADHD patients had decreased amplitude of low-frequency fluctuations (ALFF) in the right inferior frontal cortex, the bilateral cerebellum, and the vermis and increased ALFF in the right anterior cingulated cortex, left sensorimotor cortex, and bilateral brainstem [7]. An et al. found that the regional homogeneity (ReHo) method showed widely distributed differences between the two groups in the frontal-cingulate-occipital-cerebellar circuitry, while the ALFF method showed a difference only in the right occipital area [8]. Zhou et al. found that the centrality of left superior temporal gyrus (STG) decreased and the centrality of the left superior occipital lobe and right inferior parietal lobe increased in the ADHD patients [9]. Wang et al. found that the density of rich clubs among structural hub nodes was reduced in the ADHD patients including the bilateral precuneus (PCUN), the insula (INS), the caudate nucleus, the left putamen, and right calcarine [10]. In addition, abnormal hemispheric asymmetry has also been shown to be associated with common neurodevelopmental disorders, including ADHD [11–14]. He et al. conducted a comprehensive meta-analysis of a multimodal imaging dataset (rs-fMRI and structural magnetic resonance imaging) and performed lateralization analysis. The results found that forty-one percent (41%) of regions of ADHD patients had both structural and functional abnormalities in asymmetry [15]. Longarzo et al. also explored the asymmetry index significantly correlated across subjects between fMRI and power EEG in the inattention group in frontal and temporal areas [16]. Previous studies have suggested that the abnormal brain regions were closely related to ADHD symptoms. Exploratory analysis of regions allowed us to explore regional changes in the brains of patients with ADHD.

Others studies pointed that ADHD was characterized as a dysconnectivity syndrome [17], rather than a disease of isolated brain region changes [18]. Meanwhile, other ADHD-related studies also suggested that ADHD patients had distributed brain network disorders, rather than the disease with discrete regional abnormalities [19, 20]. Studies showed that the dysregulation of neural network with ADHD patients occurred not only in the dorsal attention network (DAN) and default mode network (DMN) but also in the somatosensory, motor, visual, and auditory networks [21]. With development, changes in the brain network had a greater impact on ADHD than in separate regions [22]. Lin et al. indicated that compared to healthy adolescents, the connectivity between DMN and task-positive networks increased in the ADHD patients [23]. Guo et al. found that the discriminative functional connectivity (FC) existed in the intranetwork within DMN and the internetwork between DMN and ventral attention network (VAN) in ADHD children and adults and positively correlated with ADHD symptom scores [24]. Many studies were devoted to exploring the differences in networks or independent regions between ADHD and healthy children or focusing on connections with brain prior regions of interest (ROI), but the results were closely related to the selection of seed points [25], while these researches may ignore that ADHD symptoms may affect not only changes in a single brain region or a network composed by several brain regions but also changes in the integration and separation of the whole brain.

Therefore, the research considered exploring the brain function changes of ADHD groups from the perspective of the whole brain, rather than confined to a single region or predefined network. Using the brain atlas to divide brain regions and constructing whole brain network by the graph theory were the mainstream methods. Namely, the human brain was regarded as a complex network interacting with brain regions, providing a novel way to explore brain functional mechanism from a holistic perspective [26, 27]. The results of whole brain analysis may be influenced by the segmentation of brain regions with different resolutions of brain atlas. We expected to use high nodal resolution brain atlas to detect more subtle changes. Low-resolution AAL_90 atlas was the frequently used brain atlas. The AAL_90 atlas used automatic anatomical labelling (AAL) algorithm to divide the entire cerebral cortex into 90 noncerebellar anatomic regions of interest with low resolution [28], while the AAL_1024 atlas subdivided the native AAL segmentation into 1024 micro regions of interest of approximately identical size [29]. Specifically, for generating high-resolution node scale, each node of the low-resolution native AAL atlas was subdivided into a set of contiguous micro nodes. In studies of other mental disorders, such as major depressive disorder (MDD) and amnesic mild cognitive impairment (aMCI), they got better rate of identification using the AAL-1024 atlas and detected more subtle changes in local brain function [30, 31]. Thus, this study utilized graph theory and high nodal resolution AAL_1024 atlas to analyze the brain network of ADHD groups, for finding more subtle changes of brain regions.

In addition, it was important to select different network topology properties to analyze functional network changes, because changes in topological properties of the brain network were related to abnormal cognitive development and many brain diseases [32–37]. Nodal properties were used to evaluate the contribution of important brain regions in the network. The clustering coefficient measured network separation, while the shortest path length measured network integration; small-world network was generally considered to achieve a balance between functional separation and integration [38]. Although “small-world” summarized key properties of complex networks at global and local levels of topology description, it did not provide any information about intermediate scale of the network organization, while another special topological organization, modularity, can complement the information of brain network at mesolevel [39, 40]. Modularity was topologically defined as a subset of highly interconnected nodes which were relatively sparsely connected to nodes in other modules [41]. Research showed that human brain network was modular, and modular structure of the human brain changed dynamically with aging [42]. Alexander-Bloch et al. found that in childhood schizophrenia and other developmental diseases, the modular changes in brain function network theoretically quantified the expected abnormality of the brain network [43]. Qian et al. also used graph theory to analyze the modular network topology abnormalities between the ADHD group and normal group [44], but there was no specific analysis of the differences in the modular composition of the two groups and abnormal connection changes between modules like modular studies of patients with Alzheimer disease (AD) [45]. Therefore, we focused on the alternation of modularity at mesolevel in the ADHD group and hypothesized that the abnormal modularity was the basis for the difference between the ADHD and normal groups.

Based on the above analysis, we used the AAL_1024 atlas to construct resting-state brain network with high node resolution for detecting more subtle changes of brain regions. And we analyzed the functional brain network based on the graph theory. We attempted to identify changes in functional brain network topology property between two groups at multilevel. Firstly, we analyzed changes of average network property preliminarily. Then, we attempted to find the change of node properties of the ADHD group, especially with the execution control and visual attention-related brain regions. Further, we analyzed the similarities and differences of module compositions between the two groups in detail. Finally, differential network analysis would be conducted to explore the changed network connection of the ADHD group compared with the normal group. We hypothesized that the changes of network topology property were related to the clinical symptoms of ADHD patients. We hoped that this study would provide new insights from the brain network perspective and complement the fewer modularity elaboration at the mesolevel in ADHD brain network research.

## 2. Materials and Methods

### 2.1. Participants and Data Acquisition

The research data came from Peking University, and detailed description can be downloaded from the following website: http://fcon_1000.projects.nitrc.org/indi/adhd200/. The functional images of all participants were scanned by the experimental instrument 3.0 T Siemens Trio scanner. During the experiment, participants did not require performing specific tasks but only needed to relax their minds, close their eyes tightly, and lie flat. Resting-state functional images were obtained by using a gradient-recalled echo-planar imaging (EPI) sequence with scanning parameters (repetition time (TR) = 2000 ms, echo time (TE) = 30 ms, flip angle (FA) = 90°, slice thickness = 3.5 mm, field of view (FOV) = 20 cm, and 33 slices per volume); each participant acquired a total of 240 volumes. The high-resolution T1-weighted structure images in the axial orientation were obtained by using a 3D spoiled gradient recalled (SPGR) sequence with scanning parameters (TR = 2530 ms, TE = 3.39 ms, FA = 7°, flip time = 1100 ms, slice thickness = 1.33 mm, FOV = 25.6 cm, and 128 slices per volume).

In order to obtain good information about the participants, the data were preprocessed (see Data Preprocessing), and data information of participants with large head movements and poor registration results was manually excluded. Finally, 118 participants were selected, including 61 ADHD children (i.e., 32 ADHD combined and 29 ADHD inattentive types) and 57 typically developing control children. The dimensional measures of ADHD symptoms were diagnosed by ADHD Rating Scale (ADHD-RS) IV. All participants met the conditions: (1) right-handed, (2) no history of head injury, (3) no other severe mental illness, and (4) Wechsler Intelligence Scale for Chinese Children-Revised (WISCC-R) scores greater than 80. There was no significant difference between the two groups in gender and age. Psychostimulant medications were forbidden at least 48 hours before scanning. The Research Ethics Review Board of Peking University Institute of Mental Health approved all research. Parents of each participant signed an informed consent form, and all children agreed to participate in the study. The specific age, gender, and ADHD information of participants are shown in Table 1.

### 2.2. Data Preprocessing

Data Processing Assistant for Resting-State fMRI (DPARSF) software was used to preprocess the fMRI data based on statistical parameter mapping (SPM8) and REST toolbox packages, which was part of the Data Processing and Analysis of Brain Imaging (DPABI) toolbox (http://rfmri.org/dpabi) [46]. Before processing the research data in this study, the first 10 volumes of the participants were removed to prevent the participant's state from being unstable in the initial stage of collection, and a total of 230 volumes were obtained. The main steps of data preprocessing included the following: (1) slice timing: when the instrument performed interlayer scanning, the image time of each slice had a deviation, so we needed to conduct time correction of each slice; (2) spatial realign: in order to eliminate participants with large head movements, each image of the corresponding experimental sequence was realigned with the first image according to a certain algorithm to perform head movement correction. In this study, the range was set to horizontal head movement greater than 2 mm or rotation greater than 2°; if it exceeded, remove the participant; (3) spatial normalization: in order to remove the differences in brain structures of different participants, functional images and structural images of each participant were configured. Diffeomorphic anatomical registration through exponentiated lie algebra (DARTEL) method was used to extract probability density maps of gray matter from T1 structural images and carry out spatial transformation. Then, the same parameters were used to transform the corresponding fMRI image to complete the spatial standardization; (4) remove covariates, mainly 24 head movement parameters such as cerebrospinal fluid and white matter; (5) spatial smoothing: a kernel function with a Gaussian kernel of 6 millimeters (mm) was used to smooth the standard image to improve the image signal-to-noise ratio; (6) filtering and time signal extraction. In order to eliminate high-frequency noise, the signal was band-pass filtered with a frequency of 0.01-0.08. Then, time signals were extracted according to brain regions to construct a brain network.

### 2.3. Construction of Brain Network and Property Analysis

The network was composed of many nodes and edges connecting these nodes. In this study, brain regions were used as nodes, and the connections between the brain regions were used as edges to construct the brain network. We used the AAL_1024 atlas to divide the brain, extracted the average time signal of each brain region, and calculated Pearson correlation coefficient to construct functional matrix between brain regions. Pearson correlation coefficient was a statistic that measured the degree of linear correlation between two variables; the mathematical formula was defined:

(1) $$\begin{array}{c} R_{ij}=\frac{1}{n-1}\overset{n}{\underset{i=1}{\sum }}\left(\frac{X_{i}-\bar{X}}{S_{x}}\right)\left(\frac{Y_{i}-\bar{Y}}{S_{y}}\right). \end{array}$$

Among them, *R* was the Pearson correlation coefficient, *X*_*i*_ and *Y*_*i*_ were the average time signals of two brain regions, $\bar{X}$ and $\bar{Y}$ were the mean values of the signal, *S*_*x*_ and *S*_*y*_ represented the standard deviation of the signal, and *n* was the sample size. The range of *R* fluctuated between -1 and 1. If *R* > 0, the two brain regions were in a positive correlation; if *R* < 0, the two brain regions were in a negative correlation. While *R* = 0, there was no correlation between the two brain regions. The Pearson correlation coefficient was calculated among brain regions to form the correlation coefficient matrix of brain network. In order to make the matrix conform to the normal distribution, the Fisher-*Z* transformation was performed to obtain an approximate normal distribution FC matrix. In order to distinguish whether there was a connection between brain regions, a threshold was set, and the FC matrix with weights was transformed into a binary matrix to obtain a binary network, so as to study its network properties.

In order to describe the different scales of brain network, such as the integration and separation of networks, contribution of important brain regions, and intermediate description of network, a variety of network topology properties were used in this section to quantify and describe, including the most commonly used network properties: node degree, clustering coefficient, shortest path length, local efficiency, global efficiency, small-world property, and modularity [47]. The Newman algorithm was used to perform modular analysis [48].  Table 2 explains some of the basic concepts of these properties, as well as the mathematical definition of the properties, and Table 3 shows a description of some of the relevant symbols.

### 2.4. Statistical Analysis

Brain network was binarized by setting the threshold value to study the properties of functional connection network. The selection of threshold determined the degree of sparseness of the network. If selecting a higher threshold, the network would be sparser; if selecting a lower threshold, the network would be denser. We adopted absolute threshold method to study network topology properties. In the present study, the choice of the threshold was based on the absolute threshold method [49–51]. The definition of the absolute threshold method was as follows: when the correlation coefficient between nodes in the network exceeded this threshold, it indicated that there was a connection between nodes and vice versa. In addition, the average node degree of the brain was not less than the natural logarithm of the number of nodes in the network based on the small-world property of the brain network [38]. Therefore, we established the threshold network based on the criteria of threshold selection mentioned above.

**Figure 1:**
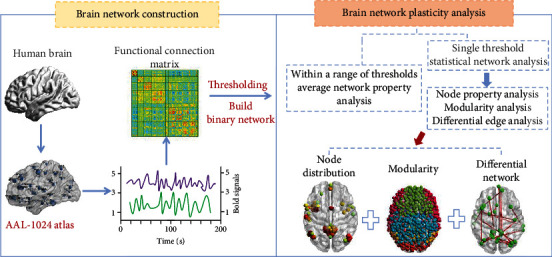
The flowchart of the analysis steps.

Therefore, we carried out the network analysis within the threshold range [0.02, 0.58] in steps of 0.02 to obtain a series of binarized brain networks. Under each threshold, we calculated the average network properties of the two groups and carried out statistical analysis. Then, choosing a reasonable single threshold, we used one-sample *t*-test to statistically analyze the FC networks of the two groups to obtain the corresponding statistical network and performed the node property analysis and modularity analysis. Finally, two-sample *t*-test method was used to statistically analyze two groups of brain networks to obtain the differential network and perform the differential edge analysis. The specific flowchart is shown in Figure 1.

## 3. Results

### 3.1. Within a Range of Threshold Average Network Property Analysis

**Figure 2:**
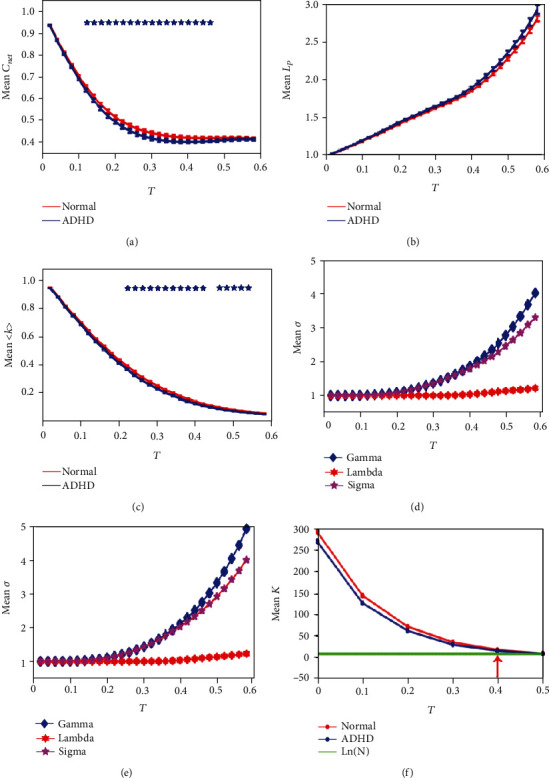
Changes of the network properties of the two groups within the threshold: (a) the average clustering coefficient (mean *C*_net_) of the two groups; (b) the average shortest path length (mean *L*_p_) of two groups; (c) the average node degree with normalization (mean <*k*>) of the two groups; (d) small-world network properties (mean *σ*) of the normal group; (e) small-world network properties (mean *σ*) of the ADHD group; (f) the average node degree (mean *k*, <*k*>) of the two groups. Blue represented the ADHD group, and red represented the normal group; the blue asterisk represented statistical significance (*p* < 0.05, Bonferroni correction), and green represented Ln(*N*); *N* was the number of network nodes.

In Figure 2(a), the average clustering coefficient of the two groups gradually decreased with the increase of the threshold. At the beginning, the average clustering coefficient of the two groups had no significant difference. Within the threshold range from 0.12 to 0.46, the average clustering coefficient of the ADHD group was obviously lower than that of the normal group (*p* < 0.05, Bonferroni correction). After the threshold of 0.46, there was no significant difference between the two groups. In Figure 2(b), the average shortest path length of the two groups increased with the increase of the threshold, but there was no significant difference in the two groups. In Figure 2(c), the average node degree of the two groups decreased with the increase of the threshold. Within the threshold range from 0.20 to 0.42 and from 0.46 to 0.54, there were significant differences between the two groups (*p* < 0.05, Bonferroni correction). The change of the average small-world network property of the two groups with the threshold is shown in Figure 2(d) (normal group) and Figure 2(e) (ADHD group). The average small-world network property of the two groups increased with the increase of the threshold, and the values of small-world network property were greater than 1. But the functional brain network of the ADHD group showed more strong small-world network property, compared with the normal group.

Through preliminary analysis of network properties within the overall threshold range, it can be judged that there were differences between the two groups of networks. Under the premise that the small-world property *σ* and the average node degree of the network 〈*k*〉 were not less than the natural logarithms of the total number of nodes *N*, two groups of networks were statistically analyzed to obtain statistical network within group and the difference network between groups. The change of the average node degree of the statistical network of the two groups within the threshold is shown in Figure 2(f). It was clearly seen that when *T* > 0.4, the average node degree 〈*k*〉 was less than the logarithm of the number of network nodes. And it was clearly seen in Figures 2(d) and 2(e) that when *T* > 0.4, *λ* was greater than 1, indicating an upward trend. In addition, when *T* was 0.4, the average clustering coefficient and average node degree of the two groups were significantly different in Figures 2(a) and 2(c). Therefore, this study used the one-sample *t*-test to get the statistical network within group and the two-sample *t*-test to get the differential network between groups when threshold *T* = 0.4.

### 3.2. Property Analysis of Statistical Network

**Figure 3:**
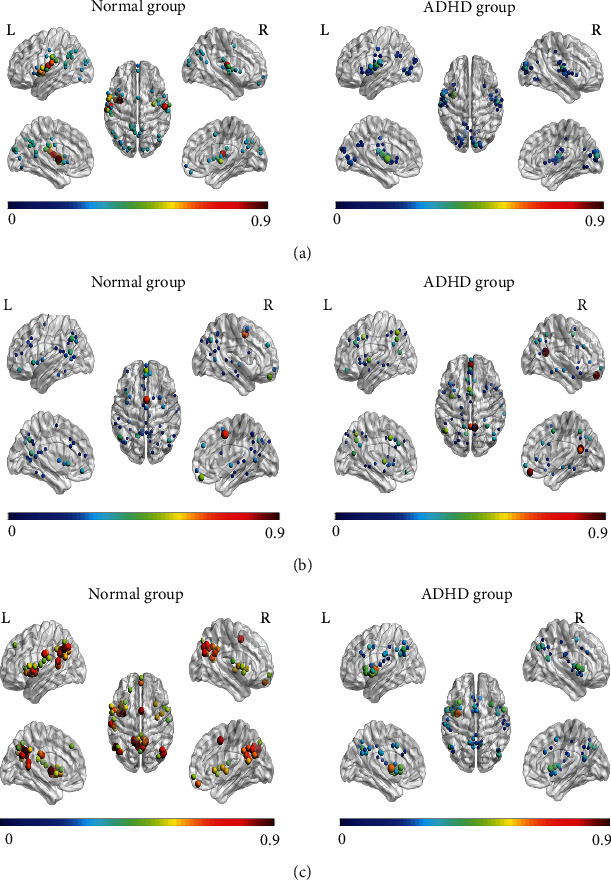
The top 50 node distributions with the highest network node properties of the two groups: (a) the top 50 node distributions with the highest node degrees of the two groups; (b) the top 50 node distributions with the highest node betweenness centrality of the two groups; (c) the top 50 node distributions with the highest global efficiency of the two groups; the left was the normal group, and the right was the ADHD group; the color and node size represented the size of the network property.

At the threshold *T* = 0.4, we performed one-sample *t*-test on the two groups to obtain the statistical network within group and analyzed the network properties, including node degree, clustering coefficient, shortest path length, betweenness centrality, global efficiency, and local efficiency. The results showed that significant differences were found in the first 50 brain regions of node degree, betweenness centrality, and global efficiency in Figure 3. At the same time, the network properties of the two groups were normalized to [0.1, 0.9] to better display the results.

#### 3.2.1. Altered Nodal Properties between Disease Statuses

In Figure 3(a), there were significant differences between the two groups of network node degrees. The top 50 node degrees of the ADHD group were significantly lower than those of the normal group, implying that the network connection of the ADHD group was relatively sparse. The ADHD group had no nodes on the anterior frontal lobe, and the distribution of nodes was relatively concentrated on the bilateral temporal occipital lobe, the INS, and posterior occipital lobe, while the distribution of nodes in the normal group was relatively more extensive.

In Figure 3(b), the nodes with betweenness centrality of the two groups were concentrated on the frontal lobe, the temporal lobe, the parietal lobe, etc. However, there were differences in the distribution of larger nodes in the two groups of networks. The larger nodes in the ADHD group were located at the right PCUN, the left gyrus rectus (REC), the inferior parietal, but supramarginal and angular gyri (IPL) and median cingulate and paracingulate gyri (DCG). However, in the normal group, the larger nodes were distributed at the right supplementary motor area (SMA), the right REC, left PCUN, left INS, and middle frontal gyrus (MFG).

In Figure 3(c), the global efficiency of the ADHD group was significantly lower than that of the normal group, indicating that the overall information transmission efficiency of the ADHD group was more inefficient. The concentrated distribution node regions of the network of two groups basically overlapped, such as the temporal lobe, left and right parietal lobe, basal ganglia, and posterior cingulate gyrus (PCG). But the ADHD group had no nodal distribution in the prefrontal lobe. The larger nodes in the normal group were mainly distributed in the left PCUN, right middle occipital gyrus (MOG), and SMA, while there were no large nodes in these areas in the ADHD group.

#### 3.2.2. Altered Modularity between Disease Statuses

**Figure 4:**
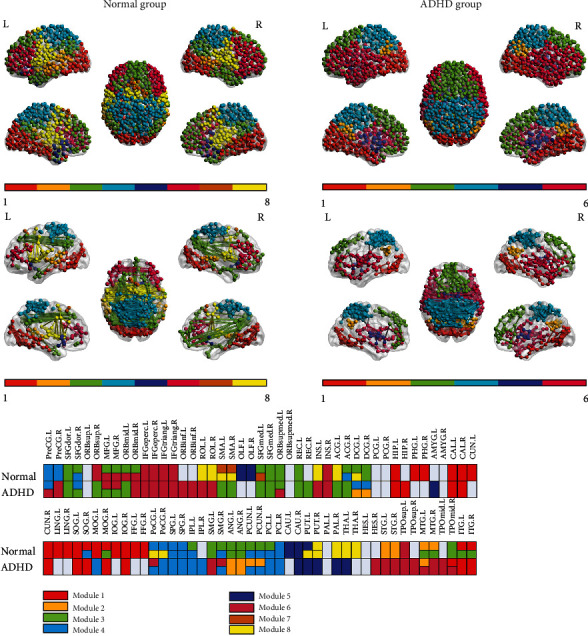
Modular distribution of two groups. The left was the normal group, and the right was the ADHD group; each color represents a module.

The results of the two groups of network modularity are shown in Figure 4; the left was the normal group, and the right was the ADHD group. The network of the ADHD group was divided into 6 modules, while that of the normal group was divided into 8 modules. Both groups had a modular structure, and the module degree *Q* was 0.672. *Q* (-0.5 to 1) was used to measure the effect of modularity division. The larger the value was, the better the modularity division was. There were similarities between modules 1 and 4 of the two groups, and the differences between the two modules were minor.

Module 1 consisted of brain regions involved in visual processing in both groups, including the superior occipital gyrus (SOG), right MOG, right inferior occipital gyrus (IOG), inferior temporal gyrus (ITG), and right cuneus (CUN). Compared with the normal group, module 1 of the ADHD group was smaller and more localized; there were fewer connections with surrounding brain areas, such as the lingual gyrus (LING), parahippocampal gyrus (PHG), and fusiform gyrus (FFG). Module 4 was mainly responsible for the somatosensory, motor, and auditory functions, including the left precentral gyrus (PreCG), postcentral gyrus (PoCG), paracentral gyrus (PCL), superior parietal gyrus (SPG), PCUN, and right supramarginal gyrus (SMG) in both groups.

However, there were obvious differences in the topological effects of certain regions and modules. For module 3, the two groups shared brain areas such as the superior frontal gyrus, dorsolateral (SFGdor), superior frontal gyrus, medial (SFGmed), left superior frontal gyrus, medial orbital (ORBsupmed), and MFG. While the ADHD group was only concentrated on the frontal lobe, it was more manifested as a short-distance connection between the frontal lobe. The normal group also included the parietal lobe and temporal lobe, such as the middle temporal gyrus (MTG) and ITG, SMG, and angular gyrus (ANG). And there were obvious long-distance connections between the frontal lobe and temporal and parietal lobe, respectively, in the normal group. For module 2, the normal group was mainly concentrated on the STG and MTG. And the ADHD group was distributed at the ANG, PCUN, median cingulate and paracingulate gyri (DCG), and left MTG. The position of module 2 of the ADHD group was displaced, and the size of the module increased.

For module 6, the normal group was concentrated on the prefrontal lobe, including the MFG, inferior frontal gyrus, triangular part (IFGtriang), and inferior frontal gyrus, opercular part (IFGoperc). However, module 6 of the ADHD group was distributed from the frontal lobe to temporal lobe, also including the SMG, right transverse temporal gyrus (TTG), STG, and MTG. And there were obvious long-distance connections between the bilateral temporal lobe in the ADHD group. Modules 7 and 8 of the normal group were mainly distributed at the SMA, thalamus (THA), and pallidum (PAL). Moreover, the newly added modules 7 and 8 of the normal group corresponded to the reorganization and separation of modules 3 and 6 of the ADHD group. In other words, the regions of the ADHD group were assembled more densely, resulting in a reduction in the total number of modules. In general, the modules in the ADHD group seemed to be more localized, resulting in fewer connections between modules.

#### 3.2.3. Differential Network Analysis

**Figure 5:**
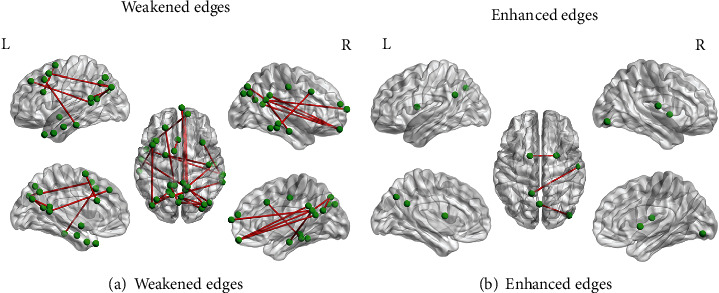
The differential edges between two groups: (a) the weakened edges of the ADHD group compared to the normal group; (b) the enhanced edges of the ADHD compared to the normal group.

The results of differential networks between the two groups are shown in Figure 5. In order to study the differences between the two groups of networks, the two-sample *t*-test method was used for statistical analysis to obtain the differential network.  Figure 5(a) shows the number of weakened edges in the ADHD group relative to the normal group. After FDR correction, 22 differential edges were obtained, mainly from the posterior occipital lobe to bilateral temporal lobe; Figure 5(b) shows that the enhanced edges of the ADHD group compared to the normal group were corrected by FDR to obtain 3 differential edges, which were mainly concentrated from the PCUN to occiput lobe. See Table 4 for details.

## 4. Discussion

This study adopted the AAL_1024 atlas to divide the brain region, constructed the brain network of the ADHD and normal groups by the graph theory, and performed plasticity analysis. We attempted to find the changes in the functional brain network topology of the ADHD and normal groups from multiple levels. From the perspective of network integration and separation at the global level, we found that the clustering coefficient of the ADHD group was significantly reduced and showed stronger small-world properties, indicating that the ADHD group had relatively sparse brain network and weakened functional connection of network. From the contribution of important brain regions at the nodal level, we observed significant differences between groups in nodal properties, especially on the frontal lobe, posterior occipital lobe, SMA, and PCG. The results of modularity at the mesolevel showed that there were short-range overconnections within module and enhanced cohesion especially in the frontal and parietal lobes, long-range underconnections between modules, and weakened coupling especially in the frontal lobe and occipital lobe in the ADHD group. We used rs-fMRI to provide reliable evidence for ADHD brain dysfunction from the perspective of whole organization and to probe large-scale neural communication in the brain from a new perspective.

### 4.1. Average Network Aberrance and ADHD-Related Changes

Based on analysis of overall threshold, although brain functional networks exhibited economical small-world topology in both groups in Figures 2(d) and 2(e), the ADHD group had stronger small-world property. In terms of average node degree and average clustering coefficient, the ADHD group was lower than the normal group in Figures 2(a) and 2(c). In one ADHD children study using the AAL_90 atlas [52], they found that the average shortest path length of the ADHD group increased compared with that of the normal group within a certain threshold range, without the difference among the two groups in the average clustering coefficient and small-world property. In another study using the Power264 atlas (data coming from Peking University of ADHD 200) [53], they found a significant difference among the groups in the average clustering coefficient but no differences in the shortest path lengths. Though the above previous studies suggested that the efficiency of local information transmission in the brain network of ADHD patients was reduced, the observed different results based on low-resolution atlas could ignore details of micro regions and thus led to the different explanations in the neural mechanisms of ADHD. Therefore, our study found that the ADHD group had lower node degree and clustering coefficient using the AAL_1024 atlas compared with the normal group, indicating that the efficiency of information transmission of local network was reduced.

The node degree indicated the interconnection between nodes, and clustering coefficient reflected the relationship between connected nodes becoming neighbors. Both node degree and clustering coefficient were indicators of the local interconnection of the network, clustering coefficient represented the efficiency of specialized processing of local information transmission, and it was considered to be the basis of the cognitive process [54]. The lower node degree and clustering coefficient indicated that normal balance of local network of the ADHD group was disturbed [55]. The functional connection of network of the ADHD group was weakened. Previous studies showed that functional connection of the ADHD group was weaker than that of the normal group, especially in the anterior cingulate gyrus, PCG, lateral prefrontal cortex, left prefrontal lobe, and THA [56, 57].

### 4.2. Nodal Properties and Differential Edges of ADHD-Related Distinctions

The results of statistical network showed that different regions among two groups were the prefrontal lobe, posterior occipital lobe, SMA, and PCG. For the node degree, it could be found that the ADHD group had no node distribution in the prefrontal lobe, and nodes in the occipital lobe were generally smaller than those of the normal group in Figure 3(a). Node degree described contribution of this node in brain network, indicating that activation of the ADHD group in the prefrontal lobe and occipital lobe was significantly weakened. The prefrontal lobe was importantly connected with advanced functions such as attention, memory, executive control, and learning; the same study also pointed out that activation of the ADHD group in the prefrontal lobe was inhibited [58, 59].

In Figure 3(b), nodes with larger betweenness centrality of the normal group were distributed in the SMA, but not in the ADHD group. This was similar to previous studies [60]. SMA was considered to be an important part of the core response inhibition system and mainly responsible for motor coordination. This may explain the hyperactivity in children with ADHD. In Figure 3(c), the results of global efficiency revealed that global efficiency of the ADHD group was generally lower than that of the normal group, especially in the SOG and PCG. Importantly, there was no node distribution in the prefrontal lobe for the ADHD group. Global efficiency determined the node information transmission efficiency in brain network, and the posterior occipital lobe was responsible for the visual processing process. Thus, the reductions of node global efficiency in these regions may lead to the lack of top-down control information between execution control and visual processing regions [61, 62].

Our results showed that there were significant differences in node degree, betweenness centrality, and global efficiency, especially in the prefrontal lobe, posterior occipital lobe, and SMA. In another study on ADHD children using the AAL_90 atlas [52], for node property, compared with the normal group, only the ADHD group showed reduced lower node efficiency in the left inferior frontal gyrus (IFG) and the left anterior cingulate cortex (ACC); our study also found that the network node efficiency of the ADHD group was much lower than that of the normal group, including the prefrontal and occipital lobe regions. Moreover, we also found the difference between groups in nodal degree and betweenness centrality. As we discussed above, these abnormal brain regions were strongly associated with ADHD symptoms. This also further proved that using high-resolution brain region atlas seemed to be able to find more local regional differences.

Statistical analysis of differential network manifested that the edges from the frontal lobe to occipital lobe were weakened in the ADHD group compared with the normal group in Figure 5(a), while the network of the ADHD group had three enhanced edges, which were mainly concentrated from the PCUN to occiput lobe in Figure 5(b). The occipital lobe was related to the processing and synthesis of visual information, which constituted a part of the selective visual attention system [63]. It indicated that even without any attention guidance, ADHD patients may pay more attention to multiple unrelated visual stimuli from the environment [64]. And enhanced occipital connections of the ADHD group were also related to poor sensory perception [65]. The PCG and PCUN were the main regions of DMN; the enhanced activity of DMN may be related to the inability of ADHD patients to concentrate. Studies showed that many mental illnesses were related to DMN [66], which was highly sensitive distinguishing between patients with mental illness and healthy individuals [67].

### 4.3. Abnormal Changes in Modularity between Disease Statuses

Resting-state functional brain network was consistently described, indicating the existence of modular organization [41, 68, 69]. A previous study pointed that modular organization contributed to all aspects of the human brain internal functional organization, such as the balance between separation and integration of brain network, while saving wiring costs and high resilience to network nodes or edge damage [70]. The results based on the modular analysis showed that the module number of the ADHD group was reduced, connections within module were tight, and connections between modules were sparse. Particularly, there were significant differences at the temporal lobe, left and right frontal lobe, and occipital lobe in Figure 4.

In the ADHD group, the bilateral prefrontal lobe modules (module 6) became larger and covered the INS and anterior temporal poles, and these regions were involved in the frontoparietal network (FPAN) [71]. The frontoparietal module in the ADHD group seemed to be “disconnected” from other modules, especially the connection with the posterior occipital module. These findings suggested that frontal parietal modules of the two groups were different. Moreover, global efficiency (especially at the frontal lobe) and local integration (corresponding to the reduction of clustering coefficient of the overall network) tended to be lower in the ADHD group. FPAN played an important role in cognitive control and decision-making, not only involving speech and working memory but also physical perception and activity inhibition. The top-down cognitive control function of the frontoparietal network helped the human brain to focus on information related to the target task and suppress the interference of irrelevant information, thereby ensuring the reasonable allocation of attention resources and effectively completing current task [72, 73]. FPAN was a key module in attention processing [74], and its topology changes were inseparable from the pathological mechanism of ADHD. Hence, this may be related to ADHD patients with learning difficulties, decreased memory, and inability to concentrate on doing things.

In addition, the ADHD group showed increased modularity in the occipital lobe (module 1), while the motor modules (modules 7 and 8 only in the normal group) disappeared. The occipital lobe was mainly involved in visual processing, and the SMA was mainly responsible for the coordination of movement [60], which may be related to hyperactivity and inability to concentrate in the ADHD group. The results of modularization also showed that the module containing the frontal and occipital lobe (module 3) existed in the normal group but disappeared in the ADHD group and became two independent modules. It further proved that there was a lack of top-down control information between the executive control region and the visual processing region in the ADHD group [61, 62].

Furthermore, the modularization results of the ADHD group also showed that there were short-range overconnectivity within module and long-range underconnectivity between modules. For example, module 3 in the normal group had a large number of long-range connections from the frontal lobe to occipital lobe, while module 3 in the ADHD group had many short-range connections in the frontal lobe. The results were consistent with the results of a review on ADHD [75]; the functional networks of ADHD patients had decreased connectivity over long-range connections but increased connectivity in short-range connections. They also echoed the ADHD group's stronger small-world property, characterized by a network with few long connections but dense local connections [38]. The above analysis results indicated that the changes of brain network topology were closely related to hyperactivity and inattention symptoms in the ADHD group. Future research could focus on these regions to find treatments that might improve the clinical symptoms of ADHD.

## 5. Conclusion

This study used the AAL_1024 atlas to construct a functional whole brain network with high nodal resolution of the ADHD group combined with the graph theory and rs-fMRI, to detect more subtle changes in brain regions and differences among groups. And we compared the network topology properties changes of the ADHD group from multiple scales with the normal group. We found that the topological changes of the ADHD group were mainly at the frontoparietal network, bilateral frontal lobe, occipital lobe, PCG, SMA, and PCUN. Overall, changes in topological property of the corresponding brain regions were closely related to the core symptoms of ADHD. Future work can explore how to effectively use brain stimulus technology to regulate the activation and connection of these brain regions, so as to improve the pathological state of ADHD patients.

## Figures and Tables

**Table 1:** Demographic and clinical characteristics of all the participants.

	Normal group (*n* = 57)	ADHD group (*n* = 61)
Gender (M/F)	57 (44/13)	61 (54/7)
Age (years)	11.24 ± 1.66	12.32 ± 1.99
ADHD index	28.05 ± 6.5	49.88 ± 8.75

**Table 2:** Mathematical definitions of complex network measures.

Measure	Formula	Definitions
Node degree	$k_{i}=\overset{N}{\underset{j}{\sum }}e_{ij}$	The greater the node degree, the more nodes connected to it, indicating that the position of the node in the network is more important
Clustering coefficient	$C_{i}=\frac{e_{i}}{k_{i}\left(k_{i}-1\right)/2}$	The clustering coefficient of nodes reflects the degree of network collectivization, which measures the relationship between nodes and their neighbors
Shortest path length	$l_{i}=\frac{1}{n-1}\underset{i\neq j\in N}{\sum }\min\left\{l_{i,j}\right\}$	The shortest path length describes the optimal path between any two nodes in the network
Betweenness centrality	$B\left(i\right)=\underset{\begin{array}{c} \text{i},\text{m}\in \text{N}\\ \text{i}\neq \text{j} \end{array}}{\sum }\frac{\varphi_{j,m}\left(i\right)}{\varphi_{j,m}}$	Betweenness centrality is defined as the number of times that the shortest path between any two nodes in the network passes through the node
Global efficiency	$E_{\text{glob}}=\frac{1}{n\left(n-1\right)}\underset{i\neq j\in N}{\sum }\frac{1}{l_{ij}}$	Global efficiency measures the global transmission capacity of the network
Local efficiency	$E_{\text{loc}}=\frac{1}{n}\underset{i\in N}{\sum }E_{\text{glob}}\left(i\right)$	Local efficiency is expressed as the average of the global efficiency of all nodes in the network
Modularity	$𝒬=\frac{1}{2m}\underset{vw}{\sum }\left[A_{vw}-\frac{k_{v}k_{w}}{2m}\right]\;\delta \left(c_{v},c_{w}\right)$	Modularity, also known as community, is defined as a collection of nodes in the network that are tightly connected inside but sparsely connected outside
Small-world network	$\gamma =\frac{C_{\text{net}}}{C_{\text{random}}},\lambda =\frac{l_{\text{net}}}{l_{\text{random}}}\approx 1,\sigma =\frac{\gamma }{\lambda }$	Small-world networks have shorter shortest path lengths and higher clustering coefficients. When *σ* > 1, it means that the network has small-world properties

**Table 3:** Basic concepts and notation.

Remarks	Basic concepts and notation
*N*	*N* is the set of all nodes in the network
*n*	*n* is the number of nodes
*e*_*ij*_	*e*_*ij*_ is the connection status between *i* and *j*: *e*_*ij*_ = 1 when link (*i*, *j*) exists (when *i* and *j* are neighbors)
min{*l*_*i*,*j*_}	min{*l*_*i*,*j*_} is defined as the number of the shortest sides between any two nodes in the network
*φ*_*j*,*m*_(*i*)	*φ*_*j*,*m*_(*i*) is the total number of the shortest paths from nodes *j* to *m* through node *i*
*φ*_*j*,*m*_	*φ*_*j*,*m*_ is the total number of the shortest paths from nodes *j* to *m*
*k*_*v*_ *k*_*w*_	*k*_*v*_ *k*_*w*_ represents the degree of nodes *v* and *w*
*δ*(*c*_*v*_, *c*_*w*_)	*δ*(*c*_*v*_, *c*_*w*_) is used to judge whether nodes *v* and *w* are in the same module; *δ*(*c*_*v*_, *c*_*w*_) = 1 when in the same module, otherwise *δ*(*c*_*v*_, *c*_*w*_) = 0

**Table 4:** Differential edge distribution.

Brain areas	MNI coordinates	Brain areas	MNI coordinates	*T* value	*p* value
Normal group < ADHD group					
Precuneus_L	(-1.3, -67.8, 38.4)	Occipital_Inf_R	(41.4, -84.2, -9)	-5.4167	*p* < 0.05
Pallidum_R	(23.3, 2.9, 1.3)	Caudate_L	(-15, 2.5, 10.7)	-5.4483	*p* < 0.05
Precuneus_L	(-12.5, -52.2, 28.5)	Rolandic_Oper_R	(55.8, -13.3, 13.5)	-5.5062	*p* < 0.05
Normal group > ADHD group					
Temporal_Inf_R	(63.6, -33.2, -15.8)	Frontal_Mid_R	(33.9, 17.0, 37.4)	4.8545	*p* < 0.05
Precuneus_R	(11.2, -45.5, 25.2)	Temporal_lobe_R	(51.9, -15.6, -21.6)	4.8822	*p* < 0.05
Frontal_Sup_L	(-21.9, 39.2, 41.2)	Precuneus_L	(-7.9, -47.8, 9.9)	4.8466	*p* < 0.05
Rectus_R	(8.4, 58.6, -18.3)	Cingulum_Mid_R	(4.8, -43.7, 32)	5.4816	*p* < 0.05
Cingulum_Ant_L	(-3.1, 21.1, 25.5)	Supp_motor_area_L	(-8.5, 4.1, 64.5)	5.1245	*p* < 0.05
Amygdala_L	(-21.4, -1.9, -11.2)	Precentral_R	(43.4, -16.5, 44.2)	5.0131	*p* < 0.05
Occipital_Sup_R	(36.1, -76.4, 44.8)	Precuneus_L	(1.8, -65.2, 25.1)	4.9316	*p* < 0.05
Precuneus_L	(-16.5, -56.6, 44.8)	Occipital_Mid_R	(32.6, -82.4, 34.4)	4.9067	*p* < 0.05
Precuneus_R	(11.2, -49.5, 25.2)	Occipital_Sup_R	(36.1, -76.4, 44.8)	5.1806	*p* < 0.05
Cuneus_L	(-2.1, -72.8, 27.7)	Occipital_Sup_R	(36.1, -76.4, 44.8)	5.6124	*p* < 0.05
Precuneus_R	(11.2, -49.5, 25.2)	Frontal_medial_R	(2.8, 69.3, 11.4)	6.1769	*p* < 0.05
Occipital_Mid_L	(-41.3, -77.4, 32.4)	Precuneus_L	(-16.5, -56.6, 15.3)	5.4060	*p* < 0.05
Rectus_R	(8.4, 58.6, -18.3)	Precuneus_R	(8.3, -54.2, 18.6)	5.4140	*p* < 0.05
Temporal_Inf_R	(64.6, -42.6, -16.4)	Parietal_Inf_L	(-34.5, -68.5, 6.3)	4.9283	*p* < 0.05
Rectus_R	(8.4, 58.6, -18.3)	Precuneus_R	(11.2, -49.5, 25.2)	6.4081	*p* < 0.05
Temporal_Mid_L	(-49.9, 6.5, -34.7)	Temporal_Mid_R	(65, -31.3, -10)	4.8834	*p* < 0.05
Temporal_pole_L	(-38, 19.4, -36.8)	Frontal_Sup_Med_R	(11.8, 59.6, 18.8)	4.9459	*p* < 0.05
Frontal_Mid_L	(-35.2, 19.5, 43.3)	Temporal_Inf_L	(-60.4, -26.3, -22.8)	4.8368	*p* < 0.05
Angular_R	(44.6, -73.3, 34.7)	Temporal_Mid_L	(-57.4, -7.9, -25.5)	6.0980	*p* < 0.05
Occipital_Mid_L	(-41.3, -77.4, 32.4)	Frontal_Mid_L	(-40.6, 13.2, 52.2)	5.0985	*p* < 0.05
Cuneus_L	(-2.1, -72.8, 27.7)	Precuneus_L	(-14.4, -47.5, 16.5)	6.1012	*p* < 0.05
Precuneus_L	(-11.8, -61.8, 38.1)	Occipital_Mid_L	(-41.3, -77.4, 32.4)	5.5756	*p* < 0.05

## Data Availability

Publicly available data were analyzed in this study. This dataset can be found here (http://fcon_1000.projects.nitrc.org/indi/adhd200/).

## References

[1] Posner J., Park C., Wang Z. (2014). Connecting the dots: a review of resting connectivity MRI studies in attention-deficit/hyperactivity disorder. *Neuropsychology Review*.

[2] Posner J., Polanczyk G. V., Sonuga-Barke E. (2020). Attention-deficit hyperactivity disorder. *Lancet*.

[3] Polanczyk G. V., Salum G. A., Sugaya L. S., Caye A., Rohde L. A. (2015). Annual research review: a meta-analysis of the worldwide prevalence of mental disorders in children and adolescents. *Journal of Child Psychology and Psychiatry*.

[4] Polanczyk G., De Lima M. S., Horta B. L., Biederman J., Rohde L. A. (2007). The worldwide prevalence of ADHD: a systematic review and metaregression analysis. *The American Journal of Psychiatry*.

[5] Raichle M. E., Mintun M. A. (2006). Brain work and brain imaging. *Annual Review of Neuroscience*.

[6] Fox M. D., Raichle M. E. (2007). Spontaneous fluctuations in brain activity observed with functional magnetic resonance imaging. *Nature Reviews. Neuroscience*.

[7] Zang Y. F., He Y., Zhu C. Z. (2007). Altered baseline brain activity in children with ADHD revealed by resting- state functional MRI. *Brain Dev*.

[8] An L., Cao Q. J., Sui M. Q. (2013). Local synchronization and amplitude of the fluctuation of spontaneous brain activity in attention-deficit/hyperactivity disorder: a resting-state fMRI study. *Neuroscience Bulletin*.

[9] Zhou M., Yang C., Bu X. (2019). Abnormal functional network centrality in drug-naïve boys with attention-deficit/hyperactivity disorder. *European Child & Adolescent Psychiatry*.

[10] Wang B., Wang G., Wang X. (2021). Rich-club analysis in adults with ADHD connectomes reveals an abnormal structural core network. *Journal of Attention Disorders*.

[11] De Guibert C., Maumet C., Jannin P. (2011). Abnormal functional lateralization and activity of language brain areas in typical specific language impairment (developmental dysphasia). *Brain*.

[12] Shaw P., Lalonde F., Lepage C. (2009). Development of cortical asymmetry in typically developing children and its disruption in attention-deficit/hyperactivity disorder. *Archives of General Psychiatry*.

[13] Eyler L. T., Pierce K., Courchesne E. (2012). A failure of left temporal cortex to specialize for language is an early emerging and fundamental property of autism. *Brain*.

[14] Altarelli I., Leroy F., Monzalvo K. (2014). Planum temporale asymmetry in developmental dyslexia: revisiting an old question. *Human Brain Mapping*.

[15] He N., Palaniyappan L., Linli Z., Guo S. (2022). Abnormal hemispheric asymmetry of both brain function and structure in attention deficit/hyperactivity disorder: a meta-analysis of individual participant data. *Brain Imaging and Behavior*.

[16] Longarzo M., Cavaliere C., Alfano V., Mele G., Salvatore M., Aiello M. (2020). Electroencephalographic and neuroimaging asymmetry correlation in patients with attention-deficit hyperactivity disorder. *Neural Plasticity*.

[17] Konrad K., Eickhoff S. B. (2010). Is the ADHD brain wired differently? A review on structural and functional connectivity in attention deficit hyperactivity disorder. *Human Brain Mapping*.

[18] Cortese S., Aoki Y. Y., Itahashi T., Castellanos F. X., Eickhoff S. B. (2021). Systematic review and meta-analysis: resting-state functional magnetic resonance imaging studies of attention-deficit/hyperactivity disorder. *Journal of the American Academy of Child and Adolescent Psychiatry*.

[19] Pruim R. H. R., Beckmann C. F., Oldehinkel M. (2019). An integrated analysis of neural network correlates of categorical and dimensional models of attention-deficit/hyperactivity disorder. *Biological Psychiatry: Cognitive Neuroscience and Neuroimaging*.

[20] Samea F., Soluki S., Nejati V. (2019). Brain alterations in children/adolescents with ADHD revisited: a neuroimaging meta-analysis of 96 structural and functional studies. *Neuroscience & Biobehavioral Reviews*.

[21] Lin H. X., Lin Q. X., Li H. L. (2021). Functional connectivity of attention-related networks in drug-naïve children with ADHD. *Journal of Attention Disorders*.

[22] Yap K. H., Manan H. A., Sharip S. (2021). Heterogeneity in brain functional changes of cognitive processing in ADHD across age: a systematic review of task-based fMRI studies. *Behavioural Brain Research*.

[23] Lin H. Y., Kessler D., Tseng W. Y. I., Gau S. S. F. (2021). Increased functional segregation related to the salience network in unaffected siblings of youths with attention-deficit/hyperactivity disorder. *Journal of the American Academy of Child and Adolescent Psychiatry*.

[24] Guo X., Yao D., Cao Q. (2020). Shared and distinct resting functional connectivity in children and adults with attention-deficit/hyperactivity disorder. *Translational Psychiatry*.

[25] Power J. D., Barnes K. A., Snyder A. Z., Schlaggar B. L., Petersen S. E. (2012). Spurious but systematic correlations in functional connectivity MRI networks arise from subject motion. *NeuroImage*.

[26] Telesford Q. K., Simpson S. L., Burdette J. H., Hayasaka S., Laurienti P. J. (2011). The brain as a complex system: Using network science as a tool for understanding the brain. *Brain Connectivity*.

[27] Biswal B., Zerrin Yetkin F., Haughton V. M., Hyde J. S. (1995). Functional connectivity in the motor cortex of resting human brain using echo- planar MRI. *Magnetic Resonance in Medicine*.

[28] Tzourio-Mazoyer N., Landeau B., Papathanassiou D. (2002). Automated anatomical labeling of activations in SPM using a macroscopic anatomical parcellation of the MNI MRI single-subject brain. *NeuroImage*.

[29] Fornito A., Zalesky A., Bullmore E. T. (2010). Network scaling effects in graph analytic studies of human resting-state fMRI data. *Frontiers in Systems Neuroscience*.

[30] Zhuo Z. Z., Mo X., Ma X. Y., Han Y., Li H. (2018). Identifying aMCI with functional connectivity network characteristics based on subtle AAL atlas. *Brain Research*.

[31] Jing B., Long Z., Liu H. (2017). Identifying current and remitted major depressive disorder with the Hurst exponent: a comparative study on two automated anatomical labeling atlases. *Oncotarget*.

[32] Cao M., Shu N., Cao Q., Wang Y., He Y. (2014). Imaging functional and structural brain connectomics in attention-deficit/hyperactivity disorder. *Molecular Neurobiology*.

[33] Cao Q., Shu N., An L. (2013). Probabilistic diffusion tractography and graph theory analysis reveal abnormal white matter structural connectivity networks in drug-naive boys with attention deficit/hyperactivity disorder. *The Journal of Neuroscience*.

[34] Zhang J., Wang J., Wu Q. (2011). Disrupted brain connectivity networks in drug-naive, first-episode major depressive disorder. *Biological Psychiatry*.

[35] Yao Z., Zou Y., Zheng W. (2019). Structural alterations of the brain preceded functional alterations in major depressive disorder patients: evidence from multimodal connectivity. *Journal of Affective Disorders*.

[36] Dai Z., Lin Q., Li T. (2019). Disrupted structural and functional brain networks in Alzheimer’s disease. *Neurobiology of Aging*.

[37] Zhou B., Dou X., Wang W. (2022). Structural and functional connectivity abnormalities of the default mode network in patients with Alzheimer’s disease and mild cognitive impairment within two independent datasets. *Methods*.

[38] Watts D. J., Strogatz S. H. (1998). Collective dynamics of ‘small-world’ networks. *Nature*.

[39] He Y., Evans A. (2010). Graph theoretical modeling of brain connectivity. *Current Opinion in Neurology*.

[40] Achard S., Bullmore E. (2007). Efficiency and cost of economical brain functional networks. *PLoS Computational Biology*.

[41] Meunier D., Lambiotte R., Bullmore E. T. (2010). Modular and hierarchically modular organization of brain networks. *Frontiers in Neuroscience*.

[42] Schlesinger K. J., Turner B. O., Lopez B. A., Miller M. B., Carlson J. M. (2017). Age-dependent changes in task-based modular organization of the human brain. *NeuroImage*.

[43] Alexander-Bloch A. F., Gogtay N., Meunier D. (2010). Disrupted modularity and local connectivity of brain functional networks in childhood-onset schizophrenia. *Frontiers in Systems Neuroscience*.

[44] Qian X., Castellanos F. X., Uddin L. Q. (2019). Large-scale brain functional network topology disruptions underlie symptom heterogeneity in children with attention-deficit/hyperactivity disorder. *NeuroImage: Clinical*.

[45] Sun Y., Yin Q., Fang R. (2014). Disrupted functional brain connectivity and its association to structural connectivity in amnestic mild cognitive impairment and Alzheimer’s disease. *PLoS One*.

[46] Chao-Gan Y., Yu-Feng Z. (2010). DPARSF: a MATLAB toolbox for “pipeline” data analysis of resting-state fMRI. *Frontiers in Systems Neuroscience*.

[47] Rubinov M., Sporns O. (2010). Complex network measures of brain connectivity: uses and interpretations. *NeuroImage*.

[48] Newman M. E. (2006). Modularity and community structure in networks. *Proceedings of the National Academy of Sciences of the United States of America*.

[49] Achard S., Salvador R., Whitcher B., Suckling J., Bullmore E. (2006). A resilient, low-frequency, small-world human brain functional network with highly connected association cortical hubs. *The Journal of Neuroscience*.

[50] He Y., Chen Z. J., Evans A. C. (2007). Small-world anatomical networks in the human brain revealed by cortical thickness from MRI. *Cerebral Cortex*.

[51] Tuleasca C., Bolton T., Régis J. (2020). Graph theory analysis of resting-state functional magnetic resonance imaging in essential tremor. *Human Brain Mapping*.

[52] Chen Y., Huang X., Wu M. (2019). Disrupted brain functional networks in drug-naïve children with attention deficit hyperactivity disorder assessed using graph theory analysis. *Human Brain Mapping*.

[53] Wang Y., Zuo C., Xu Q., Liao S., Kanji M., Wang D. (2020). Altered resting functional network topology assessed using graph theory in youth with attention-deficit/hyperactivity disorder. *Progress in Neuro-Psychopharmacology and Biological Psychiatry*.

[54] Boccaletti S., Latora V., Moreno Y., Chavez M., Hwang D. U. (2006). Complex networks: structure and dynamics. *Physics Reports*.

[55] Bullmore E., Sporns O. (2009). Complex brain networks: graph theoretical analysis of structural and functional systems. *Nature Reviews Neuroscience*.

[56] Qiu M. G., Ye Z., Li Q. Y., Liu G. J., Xie B., Wang J. (2011). Changes of brain structure and function in ADHD children. *Brain Topography*.

[57] Castellanos F. X., Margulies D. S., Kelly C. (2008). Cingulate-precuneus interactions: a new locus of dysfunction in adult attention-deficit/hyperactivity disorder. *Biological Psychiatry*.

[58] Castellanos F. X., Lee P. P., Sharp W. (2002). Developmental trajectories of brain volume abnormalities in children and adolescents with attention-deficit/hyperactivity disorder. *Jama-Journal of the American Medical Association*.

[59] Hill D. E., Yeo R. A., Campbell R. A., Hart B., Vigil J., Brooks W. (2003). Magnetic resonance imaging correlates of attention-deficit/hyperactivity disorder in children. *Neuropsychology*.

[60] Mostofsky S. H., Rimrodt S. L., Schafer J. G. (2006). Atypical motor and sensory cortex activation in attention- deficit/hyperactivity disorder: a functional magnetic resonance imaging study of simple sequential finger tapping. *Biological Psychiatry*.

[61] Li X., Jiang J., Zhu W. (2007). Asymmetry of prefrontal cortical convolution complexity in males with attention-deficit/hyperactivity disorder using fractal information dimension. *Brain and Development*.

[62] Rubia K., Halari R., Cubillo A., Mohammad A. M., Brammer M., Taylor E. (2009). Methylphenidate normalises activation and functional connectivity deficits in attention and motivation networks in medication-naive children with ADHD during a rewarded continuous performance task. *Neuropharmacology*.

[63] Mangun G. R., Hopfinger J. B., Kussmaul C. L., Fletcher E. M., Heinze H. J. (1997). Covariations in ERP and PET measures of spatial selective attention in human extrastriate visual cortex. *Human Brain Mapping*.

[64] Sripada C. S., Kessler D., Angstadt M. (2014). Lag in maturation of the brain’s intrinsic functional architecture in attention-deficit/hyperactivity disorder. *Proceedings of the National Academy of Sciences of the United States of America*.

[65] Cao X., Cao Q., Long X. (2009). Abnormal resting-state functional connectivity patterns of the putamen in medication-naive children with attention deficit hyperactivity disorder. *Brain Research*.

[66] Menon V. (2011). Large-scale brain networks and psychopathology: a unifying triple network model. *Trends in Cognitive Sciences*.

[67] Anticevic A., Cole M. W., Murray J. D., Corlett P. R., Wang X. J., Krystal J. H. (2012). The role of default network deactivation in cognition and disease. *Trends in Cognitive Sciences*.

[68] Meunier D., Lambiotte R., Fornito A., Ersche K., Bullmore E. T. (2009). Hierarchical modularity in human brain functional networks. *Frontiers in Neuroinformatics*.

[69] He Y., Wang J., Wang L. (2009). Uncovering intrinsic modular organization of spontaneous brain activity in humans. *PLoS One*.

[70] Bullmore E., Sporns O. (2009). Complex brain networks: graph theoretical analysis of structural and functional systems. *Nature Reviews. Neuroscience*.

[71] Vincent J. L., Kahn I., Snyder A. Z., Raichle M. E., Buckner R. L. (2008). Evidence for a frontoparietal control system revealed by intrinsic functional connectivity. *Journal of Neurophysiology*.

[72] Wen X., Liu Y., Yao L., Ding M. (2013). Top-down regulation of default mode activity in spatial visual attention. *The Journal of Neuroscience*.

[73] Smallwood J., Brown K., Baird B., Schooler J. W. (2012). Cooperation between the default mode network and the frontal-parietal network in the production of an internal train of thought. *Brain Research*.

[74] Markett S., Reuter M., Montag C. (2014). Assessing the function of the fronto-parietal attention network: insights from resting-state fMRI and the attentional network test. *Human Brain Mapping*.

[75] Kern J. K., Geier D. A., Sykes L. K., Geier M. R., Deth R. C. (2015). Are ASD and ADHD a continuum? A comparison of pathophysiological similarities between the disorders. *Journal of Attention Disorders*.

